# Potentially inappropriate medications and regimen complexity among elderly patients in the emergency department: Insights from a pharmacist-led medication reconciliation study

**DOI:** 10.5339/qmj.2026.8

**Published:** 2026-03-05

**Authors:** Sarah Mousavi, Mohammad Hossein Jabbary Diziche

**Affiliations:** 1 Department of Clinical Pharmacy and Pharmacy Practice, School of Pharmacy and Pharmaceutical Sciences, Isfahan University of Medical Sciences, Isfahan, Iran; 2 School of Pharmacy and Pharmaceutical Sciences, Isfahan University of Medical Sciences, Isfahan, Iran *Email: s.mousavi@pharm.mui.ac.ir

**Keywords:** Medication reconciliation, polypharmacy, inappropriate medications, hospital emergency service, aged

## Abstract

**Background:**

Polypharmacy and potentially inappropriate medications (PIMs) exposure are common in older adults, especially in emergency departments (EDs) with rapid decisions and incomplete histories. Pharmacist-led medication reconciliation improves safety but is rarely implemented in Iran.

**Objective:**

This study aimed to evaluate the prevalence and patterns of PIM use, medication regimen complexity, and associated factors among elderly patients admitted to the ED.

**Methodology:**

This cross-sectional study of patients aged 65+ years at Alzahra Hospital (October 2023–March 2024) involved medication reconciliation by pharmacy students under supervision. PIMs were identified using the 2023 American Geriatrics Society (AGS) Beers Criteria. Medication complexity and comorbidities were assessed via Medication Regimen Complexity Index (MRCI) and Charlson Comorbidity Index (CCI), with results analyzed through regression.

**Results:**

A total of 200 patients were included (mean age 78.1 ± 7.8 years; males: 109, 54.5%). Polypharmacy was observed in 104 patients (52%). The mean MRCI was 23.8 ± 13.5, and the mean CCI was 2.3 ± 1.6. PIM exposure was identified in 75 patients (37.5%), with 221 PIM prescriptions. The most frequent PIM classes were benzodiazepines (49/221, 22.2%), atypical antipsychotics (28/221, 12.7%), opioids (24/221, 10.9%), and Non-Steroidal Anti–Inflammatory Drugs (NSAIDs: 18/221, 8.1%). Clinically significant drug–drug interactions occurred in 30 patients (15%). Female sex, mild comorbidity (CCI, 1–2), and higher MRCI independently predicted PIM use.

**Conclusions:**

PIM exposure was frequent among elderly ED patients. Pharmacist-led medication reconciliation effectively identified inappropriate prescribing and complex regimens, supporting the integration of clinical pharmacists into emergency care to improve geriatric medication safety.

## 1. INTRODUCTION

The global rise in the aging population has brought increasing concern regarding the safety and appropriateness of pharmacotherapy in older adults. Due to multiple chronic comorbidities and age-related physiological changes, elderly patients are frequently prescribed several medications simultaneously, a phenomenon known as polypharmacy.^1^ While often clinically necessary, polypharmacy substantially increases the risk of drug-related problems, particularly the use of potentially inappropriate medications (PIMs).^2^ The risks are even more pronounced in the emergency department (ED),^3^ where rapid clinical decisions are made under time constraints and access to a patient’s complete medication history is often limited. In this setting, medication errors and adverse drug events (ADEs) can compromise patient safety, prolong hospital stays, contribute to readmissions, and escalate healthcare costs.^4^

Medication reconciliation, a structured pharmacist-led intervention, has been internationally recognized as a key strategy to improve medication safety during transitions of care. This process involves systematically collecting and verifying a patient’s complete medication history and comparing it with current prescriptions to identify and resolve discrepancies.^5^ The World Health Organization and other international health agencies strongly recommend its implementation, particularly in vulnerable groups such as older adults, as a large proportion of hospital medication errors and ADEs are considered preventable through reconciliation.^6^

In Iran, the importance of medication reconciliation was officially acknowledged in 2017, when it was included in the national healthcare service catalog and assigned a billing code by the Ministry of Health. However, despite this progress, widespread implementation has been limited due to operational challenges, including a shortage of trained clinical pharmacists.^7^ Consequently, older patients admitted to EDs remain at high risk for PIM exposure, inappropriate renal dosing, drug–drug interactions (DDIs), and complex medication regimens that may exceed their physiological tolerance.

Several validated tools provide structured approaches to evaluating prescribing quality in older adults. The American Geriatrics Society (AGS) Beers Criteria^8^ is widely applied to identify PIMs, while the Medication Regimen Complexity Index (MRCI)^9^ quantifies treatment burden, and the Charlson Comorbidity Index (CCI)^10^ estimates comorbidity-related risk. Prior studies in Iran and internationally have consistently shown high rates of PIM use and associations between medication complexity, comorbidity burden, and adverse drug outcomes.^11,12^ However, few studies have comprehensively examined these factors simultaneously within the high-risk setting of the ED, and there is limited evidence on the implementation of pharmacist-led medication reconciliation in this context in Iran.

Despite growing awareness of polypharmacy and PIMs in older adults, gaps remain in the literature. Most studies have been conducted in non-emergency or single-center settings, limiting their applicability to acute care. Comprehensive tools such as the Beers Criteria, MRCI, and CCI are inconsistently applied within a unified, pharmacist-led framework in EDs. Moreover, regional evidence from Iran and the Middle East is scarce, despite demographic and healthcare differences that may influence prescribing practices. Finally, few studies systematically link PIM prevalence and medication regimen complexity to patient-level factors during ED admissions, limiting the ability to guide targeted interventions and policy. This study, therefore, addresses these gaps by providing region-specific data on PIM prevalence and regimen complexity among older ED patients, highlighting the role of clinical pharmacists in optimizing pharmacotherapy and enhancing patient safety during transitions of care.

## 2. METHODOLOGY

This descriptive-analytical cross-sectional study was conducted on elderly patients aged ≥65 years who were admitted to the ED of Alzahra Hospital, Isfahan, Iran, between October 2023 and March 2024. A total of 200 patients were enrolled through consecutive sampling. Although this approach may limit generalizability, it was selected due to feasibility considerations and the exploratory nature of the study. The study protocol was reviewed and approved by the Ethics Committee of Isfahan University of Medical Sciences on August 12, 2023 (approval no.: IR.MUI.RESEARCH.REC.1402.178).

### 2.1 Inclusion criteria

- Age ≥ 65 years
- Admission to the ED of Alzahra Hospital, Isfahan, Iran

### 2.2 Exclusion criteria

- Age < 65 years
- Incomplete clinical data or inability to obtain required study information

Medication reconciliation was performed within the first 24 hours of admission by trained pharmacy students under the supervision of a clinical pharmacist. medication reconciliation was conducted using the standardized national form approved by the Ministry of Health and Medical Education (MOHME) of Iran^13^ to obtain a comprehensive medication history, including drug name, strength, dosage form, route of administration, dose, and frequency of all home medications (prescription and non-prescription), supplements, and herbal products. Medication data at discharge included both inpatient-administered drugs (from the drug Kardex^1^) and the pre-admission regimen.

Medication lists were compared with physician orders to identify discrepancies. Any discrepancy or high-risk medication was flagged in the reconciliation form under “alert medications” and reviewed by the clinical pharmacist for intervention.

Pharmacy students participating in the study received a standardized training session conducted by experienced clinical pharmacists. The training covered study objectives, inclusion/exclusion criteria, data collection procedures, documentation of Beers 2023 Criteria, MRCI, and CCI assessments, and the reconciliation workflow within the ED setting. To ensure consistency, a detailed training manual and standardized data collection forms were provided, and participants completed a hands-on practice exercise with supervision. Ongoing quality checks were performed throughout data collection, including periodic inter-rater reliability assessments and weekly reviews of collected records by the lead pharmacist to address any inconsistencies.

Demographic (age, sex, occupation), clinical (triage level, primary diagnosis, comorbidities), and laboratory data (electrolytes, serum creatinine, blood glucose, Complete Blood Count) were collected. Renal function was assessed using estimated creatinine clearance (Cockcroft–Gault equation).^14^

The study utilized validated tools, including the Beers Criteria, MRCI, and CCI, to assess medication appropriateness, regimen complexity, and comorbidity burden.

Beers Criteria (AGS 2023):^8^ Applied to identify PIMs. PIMs were classified into five categories: (i) medications to avoid;^15^ (ii) medications requiring caution; (iii) disease–drug contraindications;^9^ (iv) clinically significant DDIs; and (v) medications requiring renal dose adjustment.^8^

DDIs Analysis: All prescribed medications were screened for interactions using both the Beers 2023 Criteria and an established clinical database (Lexicomp®, Wolters Kluwer; cross-validated with Micromedex®).^16^

MRCI:^5^ A validated 65-item tool quantifying regimen complexity by dosage forms, dosing frequency, and additional instructions. Higher scores indicate more complex regimens.

CCI:^10^ Used to quantify comorbidity burden. Scores were categorized as mild (1–2), moderate (3–4), and severe (≥5).

Key terms used in this study were defined as follows.

- Discrepancy: A discrepancy is any mismatch between the patient’s home medication list, previous medical records, or reported medications and the medications documented in the current medication reconciliation form at ED admission. Discrepancies include omissions (medications the patient was taking but not recorded), duplications (repetition of the same medication), dosing errors (incorrect dose, route, or frequency), and inappropriate substitutions or omissions of prescribed therapies.
- Alert medications: Alert medications are drugs that require careful monitoring due to a narrow therapeutic index, high risk of adverse effects, or potential for severe interactions in older adults or in the study population. Examples include anticoagulants, insulin, digoxin, certain antiplatelet agents, and high-risk hypoglycemics. In this study, alert medications trigger additional verification steps or pharmacist review during reconciliation and documentation.
- Polypharmacy, defined as the concurrent use of five or more medications.

Participants were consecutively enrolled from eligible ED admissions (non-probability consecutive sampling). A priori sample size was calculated to estimate PIM prevalence with specified precision (*P* = 0.55, *Z* = 1.96, *d* = 0.068), yielding *N* ≈ 200, which was achieved during the study period.^17^ Using the formula:


N=Z1−α/22×P(1−P)/d2

$$N=Z^{\left\{2\}\_\{1-\alpha /2\right\}}\times P(1-P)/d^{\left\{2\right\}}$$

N=Z1−α/22×P(1−P)/d2

With *Z* = 1.96, *P* = 0.55, and *d* = 0.068, a minimum of 200 patients was required and achieved during the study period.

### 2.3 Statistical analysis

Data were analyzed using SPSS version 26.0 (IBM Corp., Armonk, NY). Continuous variables were expressed as mean ± standard deviation (SD) or median (IQR), as appropriate. Categorical variables were presented as frequencies and percentages.

- Comparisons between groups were performed using the independent t-test or Mann–Whitney U test for continuous variables, and chi-square or Fisher’s exact test for categorical variables.
- Linear regression analysis was used to identify predictors of medication regimen complexity (MRCI score).
- Binary logistic regression was applied to determine independent predictors of PIM exposure.
- Results were reported as β coefficients (for linear models), odds ratios^17^ with 95% confidence intervals (CIs) (for logistic models), and corresponding p-values.
- A two-tailed P-value <0.05 was considered statistically significant.

## 3. RESULTS

A total of 200 elderly patients (≥65 years) were included in this pharmacist-led medication reconciliation study conducted in the ED. The mean age of participants was 78.1 ± 7.8 years (range: 65–97), with 54.5% male. The largest proportion of patients were aged ≥85 years (*N* = 48, 24%), followed by 75 to 79 years (*N* = 46, 23%) and 70 to 74 years (*N* = 42, 21%). Detailed demographic and clinical characteristics are presented in Table 1.

The mean MRCI was 23.8 ± 13.5 (range: 3–70). The mean CCI was 2.3 ± 1.6 (range: 0–7), with 52.9% classified as mild (CCI, 1–2), 34.3% moderate (CCI, 3–4), and 12.7% severe (CCI ≥5). Fourteen percent of patients had no recorded comorbidity (Table 1).

Polypharmacy was observed in 52% of patients (*N* = 104). In total, 1239 pre-admission medications and 2049 inpatient medications were reconciled. The most frequent comorbidities were cardiovascular diseases (*N* = 137, 68.5%), diabetes mellitus (*N* = 79, 39.5%), and chronic kidney disease (*N* = 34, 17%). Mean creatinine clearance was 42.1 ± 15.3 mL/min, with 25.5% (*N* = 51) of patients requiring renal dose adjustment (Table 1).

The overall prevalence of PIMs was 37.5% (*N* = 75). In total, 221 PIM prescriptions were identified. The most frequent PIM classes were benzodiazepines (*N* = 49, 22.2%), atypical antipsychotics (*N* = 28, 12.7%), opioids (*N* = 24, 10.9%), and NSAIDs (*N* = 18, 8.1%), followed by anticholinergics, sulfonylureas, proton-pump inhibitors, and anticoagulants. The detailed distribution of PIMs by therapeutic class is shown in Table 2.

Within the category of medications requiring “use with caution,” the most common were diuretics (17.6%), followed by selective serotonin reuptake inhibitors (SSRIs) such as sertraline (5.5%) and citalopram (2.5%). Several clinically significant DDIs were identified, including concomitant use of ≥3 Central Nervous System (CNS) depressants (*n* = 19) and benzodiazepine–opioid combinations (*n* = 4). Other interactions involved spironolactone with ACEI/ARB, aspirin with NSAIDs, and digoxin with furosemide (Table 3).

Regression analyses demonstrated that higher MRCI scores were significantly associated with endocrine/metabolic diseases (β = 5.7; *P* = 0.005), higher CCI scores (β = 3.1; *P* = 0.003), polypharmacy (β = 17.9; *P* < 0.001), and PIM exposure (β = 3.19; *P* = 0.02). The results of linear regression models for predictors of MRCI are summarized in Table 4.

Binary logistic regression identified female sex (OR = 2.5, *P* = 0.008), mild comorbidity (CCI, 1–2; OR = 3.2, *P* = 0.045), and higher MRCI (OR = 1.1 per unit increase, *P* < 0.001) as independent predictors of PIM use, while age and severe comorbidity were not significant. The results of regression analysis for PIM use are presented in Table 5.

## 4. DISCUSSION

This study demonstrates a substantial burden of potentially inappropriate prescribing (PIMs) among elderly patients admitted to the ED, with 37.5% of participants receiving at least one PIM. This prevalence is broadly consistent with reports from international studies, where PIM rates among hospitalized older adults range from 30% to 55%, depending on patient characteristics, care setting, and the version of the Beers Criteria applied.^17^ Comparable figures have been reported in Southeast Asian emergency and inpatient cohorts (~39%), while higher prevalences exceeding 50% have been observed in Europe and North America, particularly in studies involving older patients with higher comorbidity burdens or longer hospital stays.^17^ The slightly lower prevalence observed in our cohort may be explained by the acute nature of ED admissions, where medication exposure may be shorter and prescribing decisions are often focused on immediate stabilization rather than chronic disease optimization.

The pattern of PIMs identified in our study—predominantly benzodiazepines, antipsychotics, opioids, and NSAIDs—mirrors findings from multiple hospital-based and ED studies.^3,15^ This consistency across diverse healthcare systems highlights persistent challenges in managing neuropsychiatric symptoms, acute pain, and inflammatory conditions in geriatric patients. Benzodiazepines remain widely prescribed despite well-established associations with delirium, falls, cognitive impairment, and fractures. Their continued use likely reflects limited access to non-pharmacological interventions, time pressure in emergency settings, and clinician familiarity with these agents. Of particular concern is the concomitant use of benzodiazepines and opioids observed in a subset of our patients, a combination known to significantly increase the risk of respiratory depression and mortality in older adults.^18^

Similarly, the frequent use of atypical antipsychotics aligns with prior studies but remains worrisome given their association with cerebrovascular events and increased all-cause mortality in older adults with dementia.^19^ In emergency settings, agitation and behavioral disturbances often necessitate rapid pharmacological control, which may partially explain their continued use despite guideline warnings.

Medications categorized as “use with caution,” particularly diuretics and SSRIs, constituted another important component of potentially inappropriate prescribing. This finding is consistent with previous literature showing that diuretics are among the most common contributors to electrolyte disturbances in older adults, especially hyponatremia, while SSRIs such as sertraline and citalopram are linked to hyponatremia and QT interval prolongation.^20^ These risks are especially salient in ED populations, where limited time and incomplete baseline data may hinder optimal monitoring and dose adjustment.

Our study also identified a high prevalence of clinically significant DDIs, with CNS polypharmacy (use of three or more CNS depressants) being the most frequent pattern. This finding is in line with previous studies demonstrating that CNS polypharmacy substantially increases the risk of sedation, falls, cognitive impairment, and functional decline in older adults. The Beers Criteria explicitly caution against such combinations, underscoring the importance of early medication review and pharmacist involvement at the point of hospital admission.^21^

Consistent with prior research, polypharmacy, comorbidity burden, and PIM exposure were key determinants of higher medication regimen complexity. Elevated MRCI scores have been repeatedly associated with poor adherence, medication errors, and increased hospitalization rates.^22^ Our findings extend existing evidence by demonstrating that PIM exposure itself contributes directly to regimen complexity, suggesting that inappropriate prescribing and regimen burden are interrelated and mutually reinforcing phenomena.

An interesting and somewhat paradoxical finding was the higher likelihood of PIM exposure among patients with mild comorbidity (CCI, 1–2) compared with those with more severe comorbidity. Similar observations have been reported in other cohorts^23^ and may reflect greater prescribing caution and closer monitoring in patients perceived as medically fragile, whereas those with fewer comorbidities may be considered “safer” candidates for higher-risk medications. This highlights the limitations of relying solely on comorbidity indices for risk stratification in geriatric prescribing.

The observed sex difference, with female patients being at higher risk of PIM exposure, aligns with previous studies.^24^ Potential explanations include sex-related differences in disease prevalence (such as depression, anxiety, and osteoporosis), pharmacokinetics, and healthcare utilization patterns, as well as greater exposure to psychotropic medications among women.

To our knowledge, this study is among the first in Iran to evaluate pharmacist-led medication reconciliation in elderly ED patients using multiple validated tools. Strengths include prospective data collection, the application of Beers Criteria 2023, MRCI, and CCI, and the simultaneous assessment of PIMs, regimen complexity, and DDIs.

### 4.1 Limitations

This study has several limitations that should be considered when interpreting the findings. First, the single-center design and consecutive sampling may limit the generalizability of the results to other EDs or healthcare settings. Second, the sample size restricted the power of subgroup analyses and may have limited the detection of smaller effect sizes. Third, only the Beers Criteria (2023) were applied to identify PIMs; the use of additional tools, such as STOPP/START, might have captured other prescribing issues. Fourth, the study focused on prescribing patterns and regimen complexity, and clinical outcomes such as adverse drug reactions, falls, readmissions, or mortality were not assessed. Finally, medication exposure was evaluated at the time of ED admission, and changes during hospitalization were not captured.

### 4.2 Clinical recommendations

Based on the study findings, several clinical implications can be highlighted. Early pharmacist-led medication reconciliation at ED admission may help identify PIMs, high-risk DDIs, and excessive regimen complexity in elderly patients. Particular attention should be given to CNS-active medications, especially benzodiazepines, antipsychotics, opioids, and their combinations. Incorporating structured geriatric prescribing tools, such as the Beers Criteria, into routine emergency care workflows may support safer prescribing decisions. Additionally, heightened vigilance is warranted even in older adults with mild comorbidity, as they may be at increased risk of inappropriate prescribing. Future multicenter studies with larger sample sizes and outcome-based follow-up are needed to further evaluate the clinical impact of pharmacist interventions in emergency settings.

## 5. CONCLUSION

This study demonstrates that pharmacist-led medication reconciliation in the ED effectively identifies medication-related risks in older adults. By systematically collecting complete medication histories and applying tools such as the Beers Criteria, MRCI, and CCI, clinical pharmacists detected PIMs, high regimen complexity, and clinically relevant DDIs. The findings highlight the critical role of pharmacists in enhancing medication safety and optimizing pharmacotherapy during transitions of care, emphasizing the impact of structured medication review on reducing potential harm in a vulnerable patient population.

## CONFLICT OF INTEREST

The authors declare no conflict of interest.

## AUTHOR CONTRIBUTIONS

SM contributed to the conceptualization and design of the study, data collection, data analysis, and drafting of the manuscript. MJ contributed to methodology development, data interpretation, and critical revision of the manuscript. SM (corresponding author) supervised the study, reviewed and edited the manuscript, and managed the submission and correspondence process. All authors read and approved the final version of the manuscript and agree to be accountable for all aspects of the work.

## Figures and Tables

**Table 1.** Demographic and clinical characteristics of patients.

Variables (*N* = 200)	
Age, mean ± SD (range)	78.1 ± 7.8 (65–97)
Age (years), *N* (%)	
65–69	31 (15.5)
70–74	42 (21)
75–79	46 (23)
80–85	33 (16.5)
≥85	48 (24)
Gender, *N* (%)	
Male	109 (54.5)
Female	91 (45.5)
MRCI, mean ± SD (range)	23.8 ± 13.5 (3–70)
Charlson Comorbidity Index, mean ± SD (range)	2.3 ± 1.6 (0–7)
No comorbidity (0)	28 (14)
Mild (1–2)	91 (52.9)
Moderate (3–4)	59 (34.3)
Severe (>5)	22 (12.7)
Number of prescribed medications	
Medication reconciliation drug, *N*	1239
Admission drugs, *N*	2049
<5	96 (48)
>5 (polypharmacy)	104 (52)
Comorbidities	
Cardiovascular	137
Diabetes	79
Chronic kidney disease	34
Gastrointestinal disorder	41
Cancer	24
COPD	25
Hyperlipidemia	24
Thyroid disorders	19
Cerebrovascular	17
Dementia	18
Benign prostate hypertrophy	15
Asthma	10
Parkinson disease	9
Arthritis	8
Liver failure	7
Others	11
ClCr (mL/min), mean ± SD	42.1 ± 15.3
Without dose adjustment, N (%)	149 (74.5)
Need to dose reduction	37 (18.5)
Need to dose increase	15 (7.5)
Disease groups (ICD-10)	
Diseases of the respiratory system	12
Diseases of the digestive system	14
Diseases of the circulatory system	52
Diseases of the nervous system	10
Endocrine, nutritional, and metabolic diseases	69
Diseases of the genitourinary system	14
Diseases of the musculoskeletal system and connective tissue	2
Neoplasms	10

**Table 2.** Frequently prescribed potentially inappropriate medications (PIMs) according to 2023 AGS Beers Criteria (*n* = 221).

Drugs listed as PIM	*n* (%)	Criteria for inappropriate use
Benzodiazepines	49	Increased risk of cognitive impairment, delirium, falls, fractures, and dependence on the elderly
Atypical antipsychotics	28	Increased risk of cerebrovascular events, mortality in dementia, and extrapyramidal symptoms
Opioids	24	Risk of sedation, respiratory depression, constipation, falls, and delirium
NSAIDs	18	Increased risk of GI bleeding, peptic ulcer disease, renal impairment, and hypertension
Anticholinergics	13	High anticholinergic burden: confusion, constipation, urinary retention, dry mouth, delirium
Sulfonylureas	14	Risk of prolonged hypoglycemia (especially with glyburide, glimepiride)
Proton pump inhibitors	13	Increased risk of C. difficile infection, bone loss, and fractures with long-term use
Anticoagulants	13	High bleeding risk in the elderly, especially without appropriate monitoring or dose adjustment
Antiplatelet agents	11	Increased risk of GI bleeding in the elderly, particularly without gastroprotection
Typical antipsychotics	7	High risk of extrapyramidal effects, sedation, and mortality in dementia
Antihistamines (1st gen)	5	Strong anticholinergic effects: confusion, sedation, urinary retention, constipation
Antidepressants (TCAs/SSRIs)	3	Risk of hyponatremia, orthostatic hypotension, anticholinergic effects, and QT prolongation
CNS agents (polypharmacy)	10	Cumulative CNS depression increases the risk of falls, delirium, and sedation.
Others	13	Agents listed in Beers’ “avoid” list due to disease–drug interactions or safer alternatives.

CNS: Central nervous system, NSAIDs: Non-steroidal Anti-Inflammatory drugs, TCA: Tri-cyclic antidepressants, SSRI: Selective serotonin reuptake inhibitor.

**Table 3.** Frequently prescribed potentially inappropriate medications (caution and drug interaction).

Drugs listed as PIM	*n* (%)	Criteria for inappropriate use
Caution		
Diuretics	39	Risk of hyponatremia, dehydration, orthostatic hypotension, and falls
Sertraline	11	Risk of SIADH/hyponatremia; caution due to increased fall risk
Citalopram	5	Dose-dependent QT prolongation; risk of arrhythmias
Empagliflozin	3	Risk of genitourinary infections, dehydration, hypotension, euglycemic ketoacidosis
Fluoxetine	3	Risk of drug–drug interactions (CYP inhibition), hyponatremia, and insomnia in the elderly
Carbamazepine	2	Risk of SIADH, hyponatremia, drug interactions, dizziness, and falls
Duloxetine	2	Increased risk of hepatotoxicity, hyponatremia, and falls
Escitalopram	1	Risk of QT prolongation and hyponatremia
Dapagliflozin	1	Risk of dehydration, urinary tract infections, and hypotension in the frail elderly
Drug interaction		
≥3 CNS agents	19	Additive CNS depression; increased risk of delirium, sedation, falls, fractures
Benzodiazepine + Opioid	4	Profound sedation, respiratory depression, overdose, and death
Aspirin + NSAIDs	1	Increased risk of gastrointestinal bleeding and renal impairment
Spironolactone + ACEI/ARB	3	High risk of hyperkalemia and arrhythmias
Fluoxetine + Warfarin	1	Increased risk of bleeding due to CYP2C9 inhibition and altered INR
NSAIDs + NSAIDs	1	Duplicate therapy increases the risk of GI bleeding and renal damage
Beta-blocker + Beta-blocker	1	Additive bradycardia and hypotension; risk of heart block
Digoxin + Furosemide	1	Risk of hypokalemia (from furosemide) potentiating digoxin toxicity (arrhythmias)

ACEI: Angiotensin converting enzyme inhibitor, ARB: Angiotensin receptor blocker, CNS: Central Nervous System, CYP: Cytochrome, GI: Gastrointestinal, NSAIDs: Non-Steroidal Anti-Inflammatory Drugs, INR: International Normalization Ratio, SIADH: Syndrome of Inappropriate Antidiuretic Hormone Secretion, QT: Q–T interval.

**Table 4.** Factors associated with MRCI in elderly patients.

Variables	Number	Linear regression	
		*β* (95% CI)	*P* value
Diseases of the respiratory system	12	−3.9 (−9.4 to 1.5)	0.16
Diseases of the digestive system	14	−2.5 (−7.7 to −2.54)	0.32
Diseases of the circulatory system	52	−0.561 (−3.8 to −2.7)	0.73
Diseases of the nervous system	10	−2.071(−7.9 to −3.7)	0.48
Endocrine, nutritional, and metabolic diseases	69	5.7 (1.7 to 9.6)	0.005
Diseases of the genitourinary system	14	2.8 (−4.4 to 10.2)	0.44
Diseases of the musculoskeletal system and connective tissue	2	2.5(−9.9 to 14.2)	0.78
Neoplasms	10	−3.1 (−9.1 to 2.7)	0.29
Charlson Comorbidity Index	-	3.1 (1.1 to 5.1)	0.003
>5 (polypharmacy)	104	17.9 (15.2 to 20.7)	0.000
Age	-	−0.125 (−0.2 to 0.04)	0.13
PIM	75	3.186 (0.5 to 5.8)	0.02

**Table 5.** Factors associated with potentially inappropriate medication use among elderly patients.

Variables	Number	Binary logistic regression	
		Odds ratio (95% CI)	P value
Diseases of the respiratory system	12	3.7 (0.6 to 21.9)	0.15
Diseases of the digestive system	14	1.9 (0.3 to 10.9)	0.44
Diseases of the circulatory system	52	2.1 (0.5 to −8.5)	0.32
Diseases of the nervous system	10	1.6 (0.2 to 10.6)	0.64
Endocrine, nutritional, and metabolic diseases	69	2.1 (0.5 to −8.4)	0.28
Diseases of the genitourinary system	14	0.74 (0.2 to −2.5)	0.63
Diseases of the musculoskeletal system and connective tissue	2	6.3 (0.3 to −14.3)	0.24
Neoplasms	10	0.59 (0.08 to 4.1)	0.6
Charlson Comorbidity Index			
Mild	119	3.2 (1.1 to 10.5)	0.47
Moderate-severe	81	1.4 (0.5 to 4.5)	
>5 (polypharmacy)	104	0.43 (0.3 to −0.8)	0.008
Age	-	1 (0.9 to 1.1)	0.7
Gender	-	0.4 (0.2 to 0.8)	0.008
MRCI	-	1.1 (1.15 to −1.05)	0.000

## Footnotes

1 Drug Kardex (also called a medication Kardex or medication cart/record) is a concise, at-a-glance nursing tool that summarizes a patient’s current medications, dosing regimens, administration times, route, frequency, and recent changes. It is used as a quick reference during rounds, admissions, discharges, and especially medication reconciliation.
